# A novel approach for estimating the nationwide incidence of renal cancer

**DOI:** 10.1186/1742-7622-11-8

**Published:** 2014-07-15

**Authors:** Andreas Stang, Christian Büchel

**Affiliations:** 1 Institut für Klinische Epidemiologie, Medizinische Fakultät, Martin-Luther-Universität Halle-Wittenberg, Halle (Saale), Saxony-Anhalt 06097, Germany; 2 School of Public Health, Department of Epidemiology Boston University, 715 Albany Street, Talbot Building, Boston, MA 02118, USA; 3 Profilzentrum für Gesundheitswissenschaften (PZG) der Medizinischen Fakultät der Martin-Luther-Universität Halle-Wittenberg, Halle (Saale), Saxony-Anhalt 06097, Germany

**Keywords:** Kidney neoplasms, Incidence, Registries, Hospitalization, Nephrectomy

## Abstract

**Background:**

The aim of this study was to provide a novel approach for estimating the incidence of renal cancer in Germany by using hospitalization data from the years 2005–2006 and to compare these estimates with incidence rates from cancer registries.

We used nationwide hospitalization data from the years 2005–2006 including 34.2 million hospitalizations. We used three definitions of potential incident renal cancer cases: 1) a main or secondary diagnosis of renal cancer and a partial or total nephrectomy; 2) a main diagnosis of renal cancer and a partial or total nephrectomy; and 3) a main diagnosis of renal cancer (without a secondary diagnosis of renal pelvis cancer) and a partial or total nephrectomy. In addition, we used cancer registry data for comparison of rates.

**Results:**

Hospitalization data to which definition 2 applied provided incidence rate estimates nearly identical to those provided by the cancer registries (when the cases registered from death certificates only were excluded). Age-standardized (European standard population) incidence rates based on hospitalization data and cancer registry data were 15.6 per 100 000 and 15.7 per 100 000 among men and 8.0 per 100 000 and 7.6 per 100 000 among women respectively. Cancer registry-based incidence rates were lower especially among those federal states with an estimated completeness of registration below 90% (Berlin and Saxony-Anhalt).

**Conclusions:**

Representative hospitalization data can be used to estimate incidence rates of renal cancer. We propose that incidence rates can be estimated by hospitalization data if 1) the primary treatment is performed during an in-hospital stay and 2) nearly all patients undergo a defined surgical procedure that is not repeated for the treatment of the same cancer. Our results may be useful for countries with no or incomplete cancer registration or for countries that use hospitalization data to provide a representative incidence of renal cancer.

## Background

Renal cancer accounts for 3.4% of all malignancies in Germany and is the most lethal urologic cancer [1]. The estimated 5-year relative survival of renal cancer patients in Germany is 74% [1]. As a rule, partial or total nephrectomy is performed before any further treatment among patients with newly diagnosed renal cancer [2]. According to an analysis of the clinical cancer registries of the Federal State of Brandenburg, Germany, for the years 2006 through 2010, 95.3% of all registered newly diagnosed renal cancers were treated by surgery within the first 6 months after diagnosis [3].

As nephrectomy is performed with general anaesthesia, it requires hospitalization. Thus, hospitalizations including a diagnosis of renal cancer and a partial, simple or radical nephrectomy may indicate incident renal cancer cases. We recently estimated the testicular cancer incidence based on hospitalizations that included a diagnosis of testicular cancer and an orchiectomy. The results were very much in line with incidence estimates provided by cancer registries in Germany [4].

Several countries either have no cancer registries or have only regional cancer registries including France, Italy, Spain, Turkey, India, China, Japan, Thailand, Brazil, Argentina, Chile, Colombia [5]. However, several of these countries use DRGs (diagnosis related groups) or self-developed DRG-like classification systems of hospitalizations for hospital reimbursement [6]. If the estimation of incidence rates of renal cancer is possible through the use of nationwide DRG hospitalization data, this approach would enable these countries to provide national and regional incidence rates despite incomplete cancer registration.

The aim of this study was to provide a novel approach for estimating the incidence of renal cancer in Germany by using hospitalization data from the years 2005–2006 and to compare these estimates with incidence rates from cancer registries.

## Material and methods

### Hospitalization data

In 2004, the DRG reimbursement system became compulsory for hospitals in Germany. According to the hospital financing law, all hospitals that are reimbursed by the DRG-system annually transfer their individual hospitalisation data to a DRG data center. Hospital stays that are reimbursed by the statutory accident insurance and hospital patient care in the ambulatory setting are not included. Furthermore, the psychiatric and psychotherapeutic departments of hospitals, military hospitals, and jail hospitals are not reimbursed by the DRG system. All hospitals that are reimbursed by the DRG system have a strong incentive to report their complete hospitalisation data. The German DRG statistics are virtually a complete record of all hospitalizations all over Germany with only a few exceptions.

The DRG data center undertakes a plausibility check of the data and generates a plausibility protocol that is sent back to the corresponding hospital. Hospitals can re-submit their corrected data files. Thereafter, the DRG data center forwards anonymised data to the Federal Bureau of Statistics. Based on confidentiality regulations (Bundesstatistikgesetz, BStatG), individual hospitalisation data are available for research purposes. Hospitalisations are anonymized which means that patients who are hospitalized more than once during the study period cannot be re-identified. By federal law, these anonymized data can be used for scientific purposes without ethical review. We were able to use the hospitalisation years 2005 and 2006 including 36.3 million hospitalisations overall.

For each hospitalization, one main diagnosis and up to 99 secondary or ancillary diagnoses coded by ICD-10 (International Classification of Diseases, 10^th^ edition) can be documented. In 2005, diagnoses were coded according to the ICD-10-GM (International Classification of Diseases, German modification) version of 2005 [7]. In 2006, the ICD-10-GM version 2006 was used [8]. The diagnosis that led to the hospitalization assessed at the end of the hospitalization is defined as the main diagnosis. Up to 100 medical procedures can be coded according to German classification of operations and procedures (OPS), a classification that represents a German version of the International Classification of Procedures in Medicine and that is updated annually by the German Institute of Medical Documentation and Information (DIMDI). In 2005 and 2006, the OPS versions for the years 2005 and 2006, respectively were used [9,10].

We used three definitions of potential incident renal cancer cases: 1) a main or secondary diagnosis of renal cancer (ICD-10: C64) and a partial or total nephrectomy (OPS: 5–553, 5–554); 2) a main diagnosis of renal cancer and a partial or total nephrectomy; and 3) a main diagnosis of renal cancer (without a secondary diagnosis of renal pelvis cancer) and a partial or total nephrectomy. The exclusion of renal pelvis cancer in definition 3 was motivated by the arbitrariness of cancer registration when the cancer report of a newly diagnosed case contains information that is too scant so that the cancer registry cannot decide whether it is a renal cancer or a renal pelvis cancer. In this case, both cancers are coded according and therefore some misclassification comes up.

Hospitalizations with a diagnosis of renal cancer but without a partial or complete nephrectomy were disregarded. The scientific use file of the DRG statistics also provides data including region of residence, age at hospital admission, and gender among others.

### Cancer registry data

The cancer registries of Hesse and Baden-Wurttemberg that were built up during our study period did not provide data. The cancer registry of North Rhine-Westphalia provided incidence data only for the administrative district of Münster. All other cancer registries including the registries from Bavaria, Bremen, Hamburg, Lower Saxony, Rhineland-Palatinate, Schleswig-Holstein, Saarland, Berlin and the new federal states including Mecklenburg-West Pomerania, Brandenburg, Saxony, Saxony-Anhalt, and Thuringia provided individual renal cancer data. We considered incidence rates derived from cancer registries with a high completeness of registration the reference standard.

The estimation of the completeness of cancer registration is undertaken by the Robert Koch-Institute in Berlin on a regular basis. This procedure starts with estimating the sex- and age-specific mortality-incidence ratios for each cancer in the federal states with a known high completeness of cancer registration (so-called reference pool of cancer registries). Under the assumption that the mortality-incidence ratios are constant across regions in Germany, these ratios and the corresponding stratum-specific mortality rates of the cancers in other federal states in Germany are used to estimate the expected number of incident cases in these regions. The ratio of the observed to the expected number of registered cases provides an estimate of registration completeness. To dampen the influence of random fluctuation, the expected and observed numbers of incident cases are modeled by log-linear regression models [11].

### Statistical analysis

The unit of analysis was the hospital admission with a diagnosis of renal cancer and a partial or total nephrectomy. We calculated crude and age-specific rates with the midyear populations of the years 2005 and 2006 as the denominators. Population data were provided by the Federal Bureau of Statistics. For the comparison across federal states, we standardized the rates using the European standard population [12]. Standard errors (SEs) of the rates were calculated by use of the binomial distribution. As federal state-wide incidence data were not available from cancer registries in North Rhine-Westphalia, Hesse and Baden-Württemberg during our study period, we excluded these states (which comprise about 42% of the German population) from the comparison of hospitalisation data-based and cancer registry-based incidence estimates to enable a one-to-one comparison between registry and hospitalization data.

For the assessment of agreement between hospitalization data and cancer registry data, the cases that were registered from death certificates only (DCO) were excluded from the cancer registry data, because such cases are likely missing from hospital records. However, according to the EUROCARE study, it should be noted that DCO cases are not necessarily a random sample of all cases as their actual survival may be much shorter than the survival of non-DCO cases [13].

For the comparison of the number of renal cancers registered by the cancer registries and estimated by the hospitalization data, we calculated the ratio of the crude incidence estimates (cancer registry) to the estimated crude incidence based on the hospitalization data. We also estimated age-specific incidence estimates based on the nationwide hospitalization data and the cancer registry data of the years 2005–2006.

## Results

From 2005 through 2006, 34.2 million hospitalizations occurred overall in Germany. Of these, a total of 25 920 hospitalizations occurred with a diagnosis of renal cancer and partial or total nephrectomy (0.08%). After the exclusion of people living outside Germany, homeless patients, and patients without known place of residence (overall n = 231), the estimated number of hospitalizations with diagnosed renal cancer and partial or total nephrectomy from 2005 through 2006 was 25 689 (median age for men: 66 years, 10^th^ and 90^th^ percentile 49 and 78 years; median age for women: 69 years, 10^th^ and 90^th^ percentile 50 and 81 years). Among these hospitalizations, 93.8% had renal cancer as the main diagnosis.

The combination of a nephrectomy and a main diagnosis of renal cancer (definition 2) produced nationwide incidence rate estimates that were closest to those provided by the cancer registries (hospital-based and cancer registry-based age-standardized rates for men: 15.6 per 100 000 and 15.7 per 100,000, respectively; hospital-based and cancer registry-based age-standardized rate for women: 8.0 per 100 000 and 7.6 per 100 000, respectively) (Table 1). When we also included hospitalization data from Hesse, North Rhine-Westphalia and Baden-Württemberg, hospitalization data-based incidence rates became slightly lower (data not shown).

**Table 1:** Comparison of the estimated crude and age-standardized incidence rates (cases per 100 000) of renal cancer in Germany from 2005 through 2006 obtained from hospitalization data to those generated by cancer registries

**Definition**	**Cases**	**Crude rate (per 100 000)**		**Age-stand. rate (per 100 000)**	
		**Rate**	**SE**	**Rate**	**SE**
Men					
Hospitalization data					
Definition 1: Main or secondary diagnosis of renal cancer, partial or total nephrectomy	9,882	21.1	0.2	16.5	0.2
Definition 2: Main diagnosis of renal cancer, partial or total nephrectomy	9,306	20.0	0.2	15.6	0.2
Definition 3: Main diagnosis of renal cancer and no simultaneous diagnosis of renal pelvis cancer, partial or total nephrectomy	9,256	19.9	0.2	15.5	0.2
Cancer registries	9,400	20.2	0.2	15.7	0.2
Women					
Hospitalization data					
Definition 1: Main or secondary diagnosis of renal cancer, partial or total nephrectomy	6,060	12.5	0.2	8.5	0.1
Definition 2: Main diagnosis of renal cancer, partial or total nephrectomy	5,691	11.7	0.2	8.0	0.1
Definition 3: Main diagnosis of renal cancer and no simultaneous diagnosis of renal pelvis cancer, partial or total nephrectomy	5,660	11.7	0.2	7.9	0.1
Cancer registries	5,604	11.6	0.2	7.6	0.1

Legend: Federal States of North Rhine-Westphalia, Hesse, and Baden-Württemberg have been excluded; SE: standard error; Death certificate only (DCO) cases were excluded from the cancer registry data.

**Figure 1:**
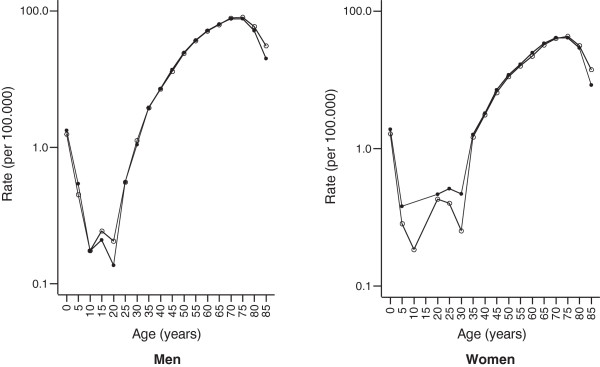
**Comparison of the estimated age-specific incidence rates (cases per 100 000) of renal cancer in Germany from 2005 through 2006 obtained from hospitalization data to those generated by cancer registries.** Hospitalization data-based incidence rates are based on definition 2; Circles: age-specific incidence of renal cancer based on cancer registries in Germany without North Rhine-Westphalia, Hesse, and Baden-Württemberg; dots: corresponding age-specific incidence of renal cancer based on DRG data; Death certificate only (DCO) cases were excluded from the cancer registry data.

**Figure 2:**
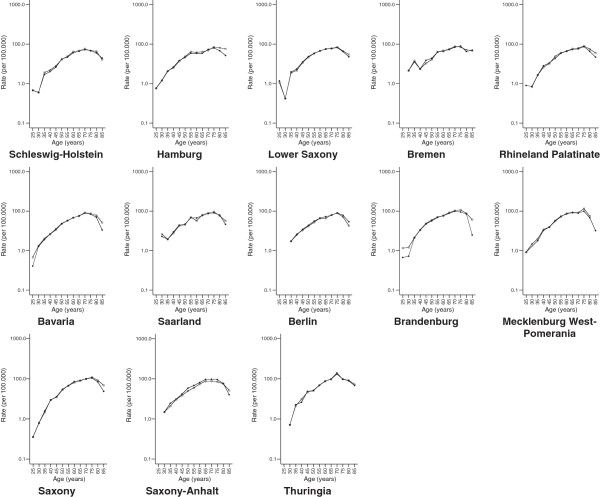
**Comparison of the estimated Federal State-specific age-specific incidence rates (cases per 100 000) of renal cancer in Germany from 2005 through 2006 obtained from hospitalization data to those generated by cancer registries among men.** Hospitalization data-based incidence rates are based on definition 2; Death certificate only (DCO) cases were excluded from the cancer registry data.

**Figure 3:**
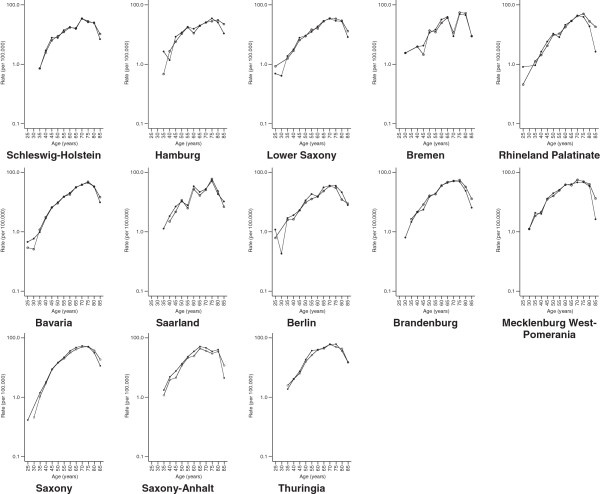
**Comparison of the estimated Federal State-specific age-specific incidence rates (cases per 100 000) of renal cancer in Germany from 2005 through 2006 obtained from hospitalization data to those generated by cancer registries among women.** Hospitalization data-based incidence rates are based on definition 2; Death certificate only (DCO) cases were excluded from the cancer registry data.

We also observed nearly identical hospitalization data-based and cancer registry-based crude rates for each federal state separately. Cancer registry-based incidence rates were lower especially among those federal states with an estimated completeness of registration below 90% (Berlin and Saxony-Anhalt) (Tables 2 and 3). The nationwide age-specific rates of the hospitalization data and cancer registry data were nearly identical for ages 30 years and more (Figure 1). The study of federal state-specific incidence age patterns produced the same results (Figures 2 and 3).

**Table 2:** Comparison of the estimated Federal State-specific crude incidence rates (cases per 100 000) of renal cancer in Germany from 2005 through 2006 obtained from hospitalization data to those generated by cancer registries among men

	**Hospitalization data**									**Cancer registry data**					**Ratio of incidence estimates**		
	**Number of cases by definition**			**Incidence rate (per 100,000)**									**Incidence rate (per 100,000) without DCO**		**Cancer registry/hospitalization data estimate**		
	**Def. 1**	**Def. 2**	**Def. 3**	**Def. 1**		**Def. 2**		**Def. 3**		**Cases**	**DCO**	**Completeness**			**Def. 1**	**Def. 2**	**Def. 3**
**State**	**N**	**N**	**N**	**Rate**	**SE**	**Rate**	**SE**	**Rate**	**SE**	**N**	**N**	**%**	**Rate**	**SE**			
**WEST**																	
Schleswig-Holstein	406	372	369	14.7	0.7	13.4	0.7	13.3	0.7	407	87	91	14.7	0.7	1.00	1.10	1.11
Hamburg	265	242	239	15.6	1.0	14.2	0.9	14.1	0.9	273	10	100	16.1	1.0	1.03	1.13	1.14
Lower Saxony	1 443	1 352	1 346	18.4	0.5	17.3	0.5	17.2	0.5	1 361	215	95	17.4	0.5	0.95	1.01	1.01
Bremen	134	128	128	20.8	1.8	19.9	1.8	19.9	1.8	129	7	92	20.1	1.8	0.97	1.01	1.01
North Rhine-Westphalia	3 166	2 976	2 960	18.0	0.3	16.9	0.3	16.8	0.3								
Adminsitrative District of Münster										449		91	17.5	0.8			
Hesse	1 130	1 061	1 052	19.0	0.6	17.8	0.5	17.6	0.5								
Rhineland-Palatina	713	673	666	17.9	0.7	16.9	0.7	16.7	0.6	726	63	100	18.2	0.7	1.02	1.08	1.09
Baden-Württemberg	1 834	1 708	1 687	17.4	0.7	16.2	0.4	16.0	0.4								
Bavaria	2 312	2 167	2 154	18.9	0.4	17.8	0.4	17.6	0.4	2 238	283	100	18.3	0.4	0.97	1.03	1.04
Saarland	233	223	223	22.8	1.5	21.8	1.5	21.8	1.5	227	6	100	22.2	1.5	0.97	1.02	1.02
Berlin	593	543	540	17.9	0.7	16.4	0.7	16.3	0.7	509	58	81	15.3	0.7	0.85	0.93	0.94
**EAST**																	
Brandenburg	679	656	654	26.8	1.0	25.9	1.0	25.8	1.0	711	46	100	28.1	1.1	1.05	1.08	1.09
Mecklenburg West-Pomerania	469	429	427	27.7	1.3	25.4	1.2	25.2	1.2	449	37	97	26.5	1.3	0.96	1.04	1.05
Saxony	1 194	1 155	1 150	28.7	0.8	27.7	0.8	27.6	0.8	1 137	95	94	27.3	0.8	0.95	0.99	0.99
Saxony-Anhalt	710	675	673	29.4	1.1	28.0	1.1	27.9	1.1	549	95	72	22.8	1.0	0.78	0.81	0.82
Thuringia	731	691	687	31.8	1.2	30.1	1.1	29.9	1.1	684	57	97	29.8	1.1	0.94	0.99	1.00
**Germany**	9 882	9 306	9 256	21.2	0.2	20.0	0.2	19.9	0.2	9 400	1 059		20.2	0.2	0.95	1.01	1.02

Legend: Definition 1: a main or secondary diagnosis of renal cancer (ICD-10: C64) and a partial or total nephrectomy (OPS: 5–553, 5–554); definition 2: a main diagnosis of renal cancer and a partial or total nephrectomy; definition 3: a main diagnosis of renal cancer (without a secondary diagnosis of renal pelvis cancer) and a partial or total nephrectomy; all rates are crude rates; SE: standard error of the rate; cases: only cases registered as non-DCO; DCO: death certificate only cases; West: West Germany, East: East Germany; age standard: European Standard Population; Germany: nationwide estimates with the exception of North Rhine-Westphalia, Hesse, and Baden-Württemberg; estimates of completeness of cancer registration were provided by the Robert Koch-Institute [11]; Death certificate only (DCO) cases were excluded from the cancer registry data.

**Table 3:** Comparison of the estimated Federal State-specific crude incidence rates (cases per 100 000) of renal cancer in Germany from 2005 through 2006 obtained from hospitalization data to those generated by cancer registries among women

	**Hospitalization data**									**Cancer registry data**					**Ratio of incidence estimates**		
	**Number of cases by definition**			**Incidence rate (per 100.000)**									**Incidence rate (per 100.000) without DCO**		**Cancer registry/hospitalization data estimate**		
	**Def. 1**	**Def. 2**	**Def. 3**	**Def. 1**		**Def. 2**		**Def. 3**		**Cases**	**DCO**	**Completeness**			**Def. 1**	**Def. 2**	**Def. 3**
**State**	**N**	**N**	**N**	**Rate**	**SE**	**Rate**	**SE**	**Rate**	**SE**	**N**	**N**	**%**	**Rate**	**SE**			
**WEST**																	
Schleswig-Holstein	256	238	233	8.8	0.6	8.2	0.5	8.0	0.5	239	77	91	8.3	0.5	0.94	1.01	1.04
Hamburg	166	154	154	9.3	0.7	8.6	0.7	8.6	0.7	150	7	100	8.4	0.7	0.90	0.98	0.98
Lower Saxony	844	777	773	10.4	0.4	9.5	0.3	9.5	0.3	795	167	95	9.8	0.3	0.94	1.03	1.03
Bremen	82	79	78	12.0	1.3	11.6	1.3	11.4	1.3	79	11	92	11.6	1.3	0.97	1.00	1.02
North Rhine-Westphalia	1 959	1 818	1 808	10.6	0.2	9.8	0.2	9.8	0.2			n,a,					
Adminsitrative District of Münster										232	n.a.	91	8.6	0.6			
Hesse	626	597	597	10.1	0.4	9.6	0.4	9.6	0.4			n,a,					
Rhineland-Palatina	432	396	394	10.4	0.5	9.6	0.5	9.5	0.5	431	59	100	10.4	0.5	1.00	1.08	1.09
Baden-Württemberg	1 032	938	931	9.4	0.3	8.6	0.3	8.5	0.3			n,a,					
Bavaria	1 432	1 345	1 338	11.2	0.3	10.6	0.3	10.5	0.3	1 361	273	100	10.7	0.3	0.96	1.01	1.02
Saarland	118	115	115	10.9	1.0	10.7	1.0	10.7	1.0	107	7	100	9.9	1.0	0.91	0.93	0.93
Berlin	359	333	328	10.3	0.5	9.6	0.5	9.4	0.5	279	98	81	8.0	0.5	0.78	0.83	0.85
**EAST**																	
Brandenburg	409	385	384	15.8	0.8	14.9	0.8	14.9	0.8	418	48	100	16.2	0.8	1.03	1.09	1.09
Mecklenburg West-Pomerania	292	276	276	17.0	1.0	16.0	1.0	16.0	1.0	286	38	97	16.6	1.0	0.98	1.04	1.04
Saxony	772	742	740	17.6	0.6	16.9	0.6	16.9	0.6	708	83	94	16.2	0.6	0.92	0.96	0.96
Saxony-Anhalt	424	405	403	16.8	0.8	16.0	0.8	15.9	0.8	337	82	72	13.3	0.7	0.79	0.83	0.84
Thuringia	474	446	444	20.0	0.9	18.8	0.9	18.7	0.9	414	47	97	17.5	0.9	0.88	0.93	0.94
**Germany**	6 060	5 691	5 660	12.5	0.2	11.7	0.2	11.7	0.2	5 604	997		11.6	0.1	0.93	0.99	0.99

Legend: Definition 1: a main or secondary diagnosis of renal cancer (ICD-10: C64) and a partial or total nephrectomy (OPS: 5–553, 5–554); definition 2: a main diagnosis of renal cancer and a partial or total nephrectomy; definition 3: a main diagnosis of renal cancer (without a secondary diagnosis of renal pelvis cancer) and a partial or total nephrectomy; all rates are crude rates; SE: standard error of the rate; cases: only cases registered as non-DCO; DCO: death certificate only cases; West: West Germany, East: East Germany; age standard: European Standard Population; Germany: nationwide estimates with the exception of North Rhine-Westphalia, Hesse, and Baden-Württemberg; estimates of completeness of cancer registration were provided by the Robert Koch-Institute [11]; Death certificate only (DCO) cases were excluded from the cancer registry data.

## Discussion

We found that the estimation of the incidence of renal cancer based on hospitalization data produces incidence rate estimates nearly identical to those based on cancer registries with a high completeness of registration after exclusion of DCO cases. The observation of lower incidence rates from cancer registries than from hospitalization data in the Federal State of Saxony-Anhalt and Berlin is plausible, as the cancer registries of these states are known to have been less complete (below 90%) than the other cancer registries during the study period.

Several medical factors may influence the hospital-based incidence estimates. First, patients with renal cancer might undergo a partial nephrectomy more than once. We were not able to identify these patients. However, this proportion is expected to be very low because patients undergoing surgery for renal cancer undergo intraoperative histological assessment to verify that the complete tumor has been removed (so-called R0 resection). If R0 is not verified, the surgeon increases the amount of resection until R0 is reached during the same surgery. Therefore, a partial or complete nephrectomy during a further hospital stay is an unlikely event. Second, patients with metastastic renal cancer at primary treatment might undergo surgery for debulking (nephrectomy for tumor reduction). However, these patients were detected by our algorithm as we searched for nephrectomy of any kind. Furthermore, these patients might later undergo surgery of metastases. However, these surgeries have different procedures codes than those for partial or total nephrectomy. Third, patients may be too ill to undergo nephrectomy. These patients cannot be detected by our algorithm. However, according to an analysis of the clinical cancer registries of the Federal State of Brandenburg, Germany, for the years 2006 through 2010, 95.3% of all registered newly diagnosed renal cancers were treated by surgery within the first 6 months after diagnosis [3]. However, a part of the remaining patients undergo later surgery (e.g. debulking). Therefore, the 4.5% of patients who missed surgery is most likely an overestimate. In addition, it is likely that some renal cancer reports to the cancer registry of Brandenburg were false-negative or incomplete in terms of reported surgery information. Fourth, although a rare event, patients with a renal cancer who underwent surgical treatment may have developed a further renal cancer (secondary primary) during our study period. Therefore, the proportion of patients that is missed will be small. The age-specific comparison of incidence based on hospitalization data and cancer registry data reveals that especially renal cancer patients at very high age (85+ years) may be underdetected by the hospitalization-based approach.

There are also quality-related factors that may influence the agreement between cancer registry-based incidence rates and hospitalization data-based incidence rates of renal cancer. The agreement is influenced by the recording practices and quality of coding (diagnostic codes and procedure codes) used for the hospital stays. Many countries in Europe, the United States, and Australia that use DRGs or self-developed DRG-like classification systems for hospital reimbursement [6] have a strong financial incentive to code diagnoses and procedures that are relevant to reimbursement. Furthermore, the agreement is influenced by the degree of completeness of cancer registration. As in Germany, there are many regional cancer registries that provide incidence estimates based on incomplete registration. Especially the new federal states of Germany suffer from registration incompleteness which explains higher incidence estimates based on hospitalization data than on registry data.

In Europe, the highest incidence rates of renal cancer are observed in Central and Eastern Europe [14]. The higher incidence of and mortality [15] from renal cancer in East than in West Germany among both men and women has been observed for decades and has prompted a population-based multicenter case–control study in West and East Germany between 1991 and 1995. The study found that substantial exposure to metals and solvents were associated with an increased risk of renal cancer [16]. The authors of that study hypothesized that the East–west difference in renal cancer incidence in Germany may be explained by lower technological standards of industrial production in the former German Democratic Republic [16]. The lower incidence rates in West Germany may explain why the hospitalization data based incidence rate for Germany decreased when we added the federal states of Hesse, North Rhine-Westphalia and Baden-Württemberg, all located in West Germany.

## Conclusions

In conclusion, hospitalization data can be used to estimate incidence rates of renal cancer. We propose that incidence rates can be estimated by hospitalization data if 1) the primary treatment is performed during an in-hospital stay and 2) nearly all patients undergo a defined surgical procedure that is not repeated for the treatment of the same cancer. However, in contrast to cancer registries, German hospitalization data cannot be used for estimating histology-specific incidence rates as hospitalization data do not include histology codes. We have provided empirical evidence that incidence rates for testicular cancer can be validly estimated by hospitalization data previously [4] and for renal cancer in this report. Another cancer eligible for this approach is gallbladder cancer that is typically treated in-hospital by removal of the gallbladder. Our results may be useful for countries with no or incomplete cancer registration or for countries that use hospitalization data to estimate incidence of renal cancer.

## Abbreviations

BStatG: Federal Statistics Law (Bundestatistikgesetz); DRG: Diagnosis-related group; ICD: International classification of diseases; SE: Standard error of the rate.

## Competing interests

None of the authors declared any conflict of interest.

## Authors’ contributions

AS: wrote the study protocol, regulated the access to the DRG and cancer registry data, supervised the statistical analysis and programming, programmed several parts of the analyses presented, interpreted the results and wrote the manuscript. CB: performed some of the statistical analyses, programmed some parts of the analyses presented, interpreted the results and wrote the manuscript. Both authors read and approved the final manuscript.
